# Supplementary material for: The Immune Profile of Major Dysmood Disorder: Proof of Concept and Mechanism Using the Precision Nomothetic Psychiatry Approach
Source: Cells. 2022 Mar 31;11(7):1183. doi: 10.3390/cells11071183 (PMC8997660; doi:10.3390/cells11071183)
Supplement: Supplementary file 1 [file cells-11-01183-s001.zip › cells-1652950-supplementary.pdf]

Supplementary Materials

The Immune Profile of Major Dysmood Disorder: Proof of Concept and Mechanism Using the Precision Nomothetic Psychiatry Approach

Michael Maes [1,2,3,*], Muanpetch Rachayon [1], Ketsupar Jirakran [1,4], Pimpayao Sodsai [5,6], Siriwan Klinchanhom [5,6], Piotr Gałecki [7], Atapol Sughondhabirom [1] and Agnieszka Basta-Kaim [8]

1 Department of Psychiatry, Faculty of Medicine, Chulalongkorn University and King Chulalongkorn Memorial Hospital, The Thai Red Cross Society, Bangkok 10330, Thailand; muanpetch.mp@gmail.com (M.R.); 6271001030@student.chula.ac.th (K.J.); atapol.s@gmail.com (A.S.)
2 IMPACT Strategic Research Center, Barwon Health, Deakin University, Geelong VIC 3220, Australia
3 Department of Psychiatry, Medical University of Plovdiv, 4002 Plovdiv, Bulgaria
4 Maximizing Thai Children's Developmental Potential Research Unit, Department of Pediatrics, Faculty of Medicine, Chulalongkorn University, Bangkok 10330, Thailand
5 Center of Excellence in Immunology and Immune-Mediated Diseases, Department of Microbiology, Faculty of Medicine, Chulalongkorn University, Bangkok 10330, Thailand; yokpim@gmail.com (P.S.); siriwanklinchanhom@gmail.com (S.K.)
6 Department of Microbiology, Division of Immunology, Faculty of Medicine, Chulalongkorn University, Bangkok 10330, Thailand
7 Department of Adult Psychiatry, Medical University of Lodz, 91-229 Lodz, Poland; piotr.galecki@umed.lodz.pl
8 Laboratory of Immunoendocrinology, Department of Experimental Neuroendocrinology, Polish Academy of Sciences, 31-343 Kraków, Poland; basta@if-pan.krakow.pl
* Correspondence: dr.michaelmaes@hotmail.com

Supplementary Table S1. Cytokine, chemokines and growth factors examined in the current study

Protein Abbreviations	Protein Name	Gene Symbol	Number of Samples that Lower than Sensitivity of the assay (%)
IL-1β	Interleukin-1β	IL1B	0
IL-1RA	Interleukin-1 receptor antagonist	IL1RN	0
IL-2	Interleukin-2	IL2	0
IL-4	Interleukin-4	IL4	0
IL-5	Interleukin-5	IL5	0
IL-6	Interleukin-6	IL6	0
IL-7	Interleukin-7	IL7	62.5
CXCL8	C-X-C motif chemokine ligand 8 (IL-8)	CXCL8	0
IL-9	Interleukin-9	IL9	0
IL-10	Interleukin-10	IL10	0
IL-12	Interleukin-12	IL12	0.5
IL-13	Interleukin-13	IL13	30
IL-15	Interleukin-15	IL15	0
IL-17	Interleukin-17	IL17	0
CCL11	Eotaxin	CCL11	0
FGF2	Fibroblast growth factor 2, Basic fibroblast growth factor	FGF2	0
G-CSF	Granulocyte Colony Stimulating Factor, Colony Stimulating Factor 3 (Granulocyte)	CSF3	0
GM-CSF	Granulocyte-macrophage colony-stimulating factor, Colony-stimulating factor 2	CSF2	0
IFN-γ	Interferon-γ	IFNG	0
CXCL10	C-X-C motif chemokine ligand 8, Interferon gamma-induced protein 10 (IP10)	CXCL10	0
CCL2	C-C Motif Chemokine Ligand 2 (MCP1)	CCL2	0

CCL3 / MIP-1α	Macrophage inflammatory protein-1 alpha, C-C Motif Chemokine Ligand 3	CCL3	0
PDGF	Platelet Derived Growth Factor Subunit A	PDGFA	0
CCL4 / MIP-1β	C-C Motif Chemokine Ligand 4, Macrophage Inflammatory Protein 1-Beta, Lymphocyte Activation Gene 1 Protein	CCL4	0
CCL5 /RANTES	C-C Motif Chemokine Ligand 5, Regulated Upon Activation, Normally T-Expressed, And Presumably Secreted	CCL5	0
TNF-α	Tumor Necrosis Factor-Alpha	TNF	0
VEGF	Vascular Endothelial Growth Factor	VEGFA	0

Adapted from: [14].

Supplementary Table S2. Description of the immune profiles used in this study

Immune Profile	Members
**M1 macrophage**	IL-1β, sIL-1RA, IL-6, TNF-α, CXCL8, CCL3
**T helper-1**	IL-2, IFN-γ, IL-12
**T helper-2**	IL-4, IL-5, IL-9, IL-13
**T helper-17**	IL-6, IL-17
**IRS**	IL-1β, IL-6, TNF-α, CXCL8, CCL3, IL-2, IFN-γ, IL-12, IL-17, IL-15, G-CSF, GM-CSF, CXCL10, CCL5, CCL2
**CIRS**	IL-4, IL-10, sIL-1RA
**Neurotoxicity**	IL-1β, IL-6, TNF-α, CXCL8, CCL3, IL-2, IFN-γ, IL-12, IL-17, CXCL10, CCL11, CCL5, CCL2
**Growth factors**	FGF, PDGF, VEGF
**T cell growth**	IL4, IL9, IL12, GM-CSF, IL-15

IRS: immune-inflammatory response system; CIRS: compensatory immunoregulatory system

Adapted from: [14].
